# Supplementary figures and images for: The NDNF-like factor Nord is a Hedgehog-induced extracellular BMP modulator that regulates Drosophila wing patterning and growth
Source: eLife. 2022 Jan 17;11:e73357. doi: 10.7554/eLife.73357 (PMC8856659; doi:10.7554/eLife.73357)

Figure 3-source data 1\_Uncropped western blot for Figure 3

3B

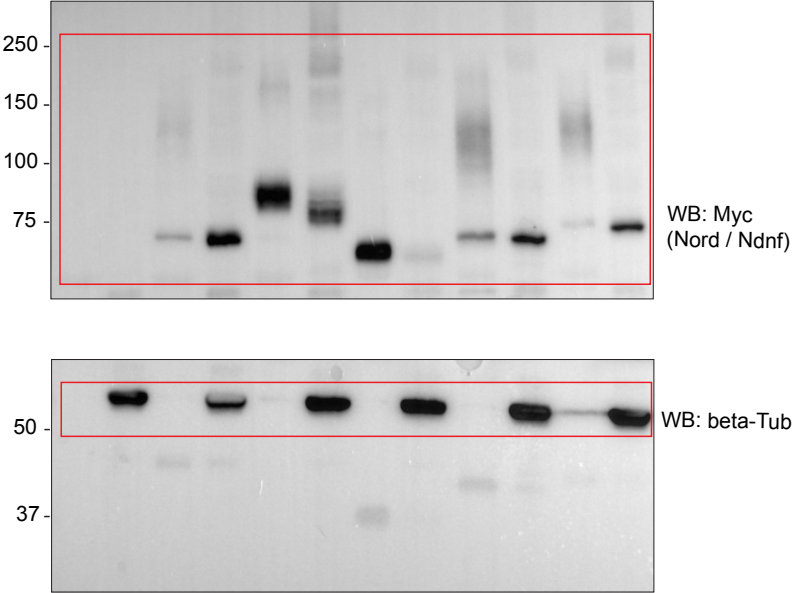

Supplement: Figure 3—source data 1. [file elife-73357-fig3-data1.pdf]

Figure 8-source data 1\_Uncropped western blot for Figure 8

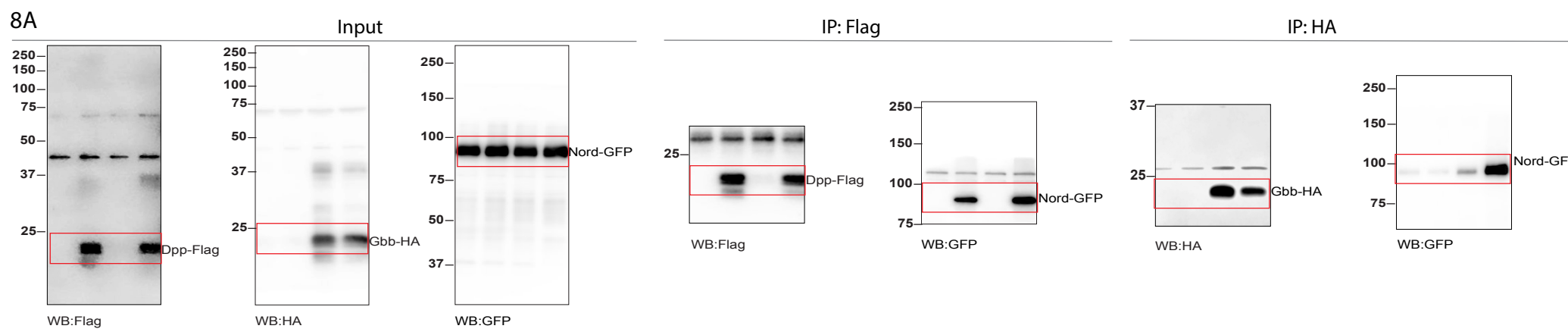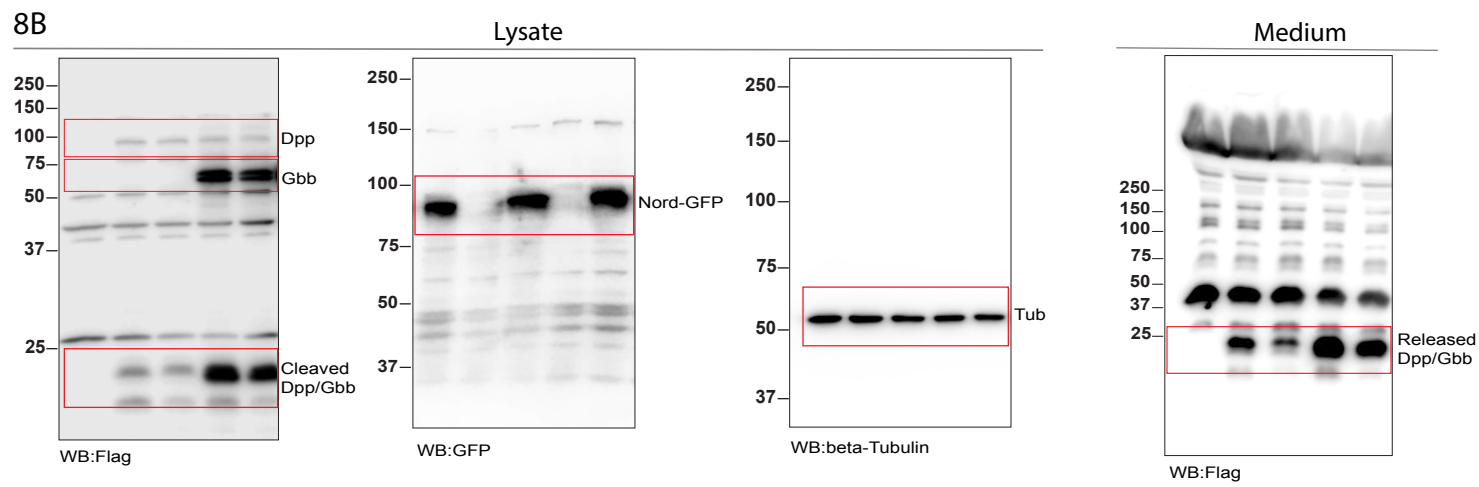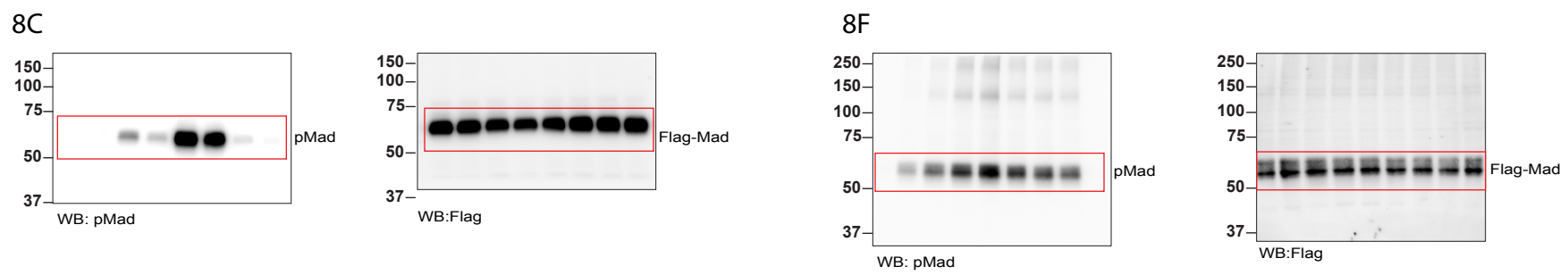

Supplement: Figure 8—source data 1. [file elife-73357-fig8-data1.pdf]
