# Supplementary material for: The NDNF-like factor Nord is a Hedgehog-induced extracellular BMP modulator that regulates Drosophila wing patterning and growth
Source: eLife. 2022 Jan 17;11:e73357. doi: 10.7554/eLife.73357 (PMC8856659; doi:10.7554/eLife.73357)
Supplement: Supplementary file 2. [file elife-73357-supp2.pdf]

Genotype		Male (n)	Female (n)
Mi{MIC}nord^{MI06414}/Df(2R)BSC770	Normal	81	72
	Ectopic PCV	0	0
Mi{MIC}nord^{MI06414}/Df(2R)BSC356	Normal	50	21
	Ectopic PCV	7	47
Mi{MIC}nord^{MI06414}/Df(2R)BSC155	Normal	46	19
	Ectopic PCV	12	81
Mi{MIC}nord^{MI06414}/Df(2R)BSC780	Normal	33	12
	Ectopic PCV	19	57
Mi{MIC}nord^{MI06414}/Df(2R)BSC603	Normal	67	68
	Ectopic PCV	0	0
Mi{MIC}nord^{MI06414}/Df(2R)ED4061	Normal	65	51
	Ectopic PCV	0	0

Supplementary file 2. Quantification of the PCV phenotypes associated with *trans*-heterozygotic combinations of *Mi{MIC}nord^{MI06414}* and tested deficiency lines
