# Supplementary material for: The NDNF-like factor Nord is a Hedgehog-induced extracellular BMP modulator that regulates Drosophila wing patterning and growth
Source: eLife. 2022 Jan 17;11:e73357. doi: 10.7554/eLife.73357 (PMC8856659; doi:10.7554/eLife.73357)
Supplement: Supplementary file 3. [file elife-73357-supp3.docx]

| Figure 2 | *(B)*   - *y, w; Mi{PT-GFSTF.2}nord^MI06414-GFSTF.2^ /+*   *(C)*   - *y, w, hs-FLP, UAS-mCD8-GFP/+; actin>y+>Gal4 / Mi{PT-RFPHA.2}nord^MI06414-RFPHA.2^; Tm6B/+*   *(D)*   - *y, w, hs-FLP, UAS-mCD8-GFP/+; actin>y+>Gal4 / Mi{PT-RFPHA.2}nord^MI06414-RFPHA.2^; UAS-SmoGlu/+* |
| --- | --- |
| Figure 3 | *(D)*   - *y, w; Mi{PT-GFSTF.2}nord^MI06414-GFSTF.2^ / +* - *y, w;*   *(E)*   - *y, w, hs-FLP/+; actin>y+>Gal4 / +; UAS-DsRed / UAS-Nord-HA-GFP* - *y, w, hs-FLP/+; actin>y+>Gal4 / +; UAS-DsRed /Tm6B* |
| Figure 4 | *(B, C)*   - *y, w;* - *y, w; Mi{MIC}nord^MI064^/ Mi{MIC}nord^MI0641^*   *(D, E)*   - *y, w;* - *y, w; Mi{MIC}nord^MI064^ / Mi{MIC}nord^MI0641^* - *w; nord^22A^ / nord^22A^* - *w; nord^22A^ / Df(2R)BSC155* - *y, w; Mi{MIC}nord^MI064^ / Df(2R)BSC155* |
| Figure 5 | *(A)*   - *y, w; Mi{PT-GFSTF.2}nord^MI06414-GFSTF.2^ / +*   *(B-E)*   - *y, w;* - *y, w; Mi{MIC}nord^MI064^ / Mi{MIC}nord^MI0641^* |
| Figure 7 | *(A-D)*  *w; nub-Gal4 / +; UAS-GFP / +*  *w; nub-Gal4 / +; UAS-Nord-HA-GFP / +* |
| Figure 8 | - *w; en-Gal4, tub-Gal80^ts^ / +; UAS-GFP / +* - *w; en-Gal4, tub-Gal80^ts^ / +; UAS-GFP / UAS-GFP* - *w; en-Gal4, tub-Gal80^ts^ / UAS-GFP; UAS-GFP / UAS-GFP* - *w; en-Gal4, tub-Gal80^ts^ / +; UAS-Nord-HA-GFP / +* - *w; en-Gal4, tub-Gal80^ts^ / UAS-Nord-HA-GFP; UAS-Nord-HA-GFP / +* - *w; en-Gal4, tub-Gal80^ts^ / UAS-Nord-HA-GFP; UAS-Nord-HA-GFP / UAS-Nord-HA-GFP* - *w; tub-Gal80^ts^ / UAS-Nord-HA-GFP; hh-Gal4 / +* - *w; tub-Gal80^ts^ / UAS-Nord-HA-GFP; hh-Gal4 / UAS-Nord-HA-GFP* |
| Figure 1-figure supplement 1 | *(B)*   - *w; ptc-Gal4/+; UAS-mCD8-GFP/+*   *(C)*   - *w; UAS-mCD8-GFP/+; hh-Gal4/+* |
| Figure 2-figure supplement 3 | *(A)*   - *w; Mi{PT-GFSTF.2}nord^MI06414-GFSTF.2^/+; hs-Gal4 /+*   *(B)*   - *w; Mi{PT-GFSTF.2}nord^MI06414-GFSTF.2^/+; hs-Gal4 / UAS-Hh*   *(C)*   - *w; Mi{PT-GFSTF.2}nord^MI06414-GFSTF.2^/+; hs-Gal4 /UAS-Ptc* |
| Figure 4-figure supplement 2 | *(A, B)*   - *w; nord^22A^/nord^3D^* - *w; nord^3D^/+* - *w;* |
| Figure 4-figure supplement 5 | *(A)*   - *w; nord^3D^ / +* - *w; nord^3D^ / Df(2R)BSC155*   *(B)*   - *w; nord^22A^ / +* - *w; nord^22A^ / Df(2R)BSC155* |
| Figure 4-figure supplement 6 | *(A)*   - *y, w; Mi{MIC}nord^MI064^ / +* - *y, w; Mi{MIC}nord^MI064^ / Df(2R)BSC155*   *(B)*   - *w; nord^22A^ / +* - *w; nord^22A^ / Df(2R)BSC155* |
| Figure 5-figure supplement 1 | - *y, w; Mi{PT-GFSTF.2}nord^MI06414-GFSTF.2^ / +* |
| Figure 5-figure supplement 2 | *(A, B)*   - *y, w;* - *y, w; Mi{MIC}nord^MI064^ / Mi{MIC}nord^MI0641^* |
| Figure 5-figure supplement 3 | - *w, A9-Gal4 / +; UAS-GFP / +* - *w, A9-Gal4 / +; UAS-nordRNAi-1 / +* - *w, A9-Gal4 / +; UAS-nordRNAi-2 / +* - *w; ptc-Gal4 / UAS-GFP* - *w; ptc-Gal4 / UAS-nordRNAi-1* - *w; ptc-Gal4 / UAS-nordRNAi-2* - *w; UAS-GFP /+; hh-Gal4 / +* - *w; UAS-nordRNAi-1 /+; hh-Gal4 / +* - *w; UAS-nordRNAi-2 /+; hh-Gal4 / +* |
| Figure 5-figure supplement 4 | - *w, A9-Gal4 / +; UAS-GFP / +* - *w, A9-Gal4 / +; UAS-nordRNAi-1 / +* - *w, A9-Gal4 / +; UAS-nordRNAi-2 / +* - *w; ptc-Gal4 / UAS-GFP* - *w; ptc-Gal4 / UAS-nordRNAi-1* - *w; ptc-Gal4 / UAS-nordRNAi-2* - *w; UAS-GFP /+; hh-Gal4 / +* - *w; UAS-nordRNAi-1 /+; hh-Gal4 / +* - *w; UAS-nordRNAi-2 /+; hh-Gal4 / +* |
| Figure 7- figure supplement 1 | - *w; nub-Gal4 / +; UAS-GFP / +* - *w; nub-Gal4 / +; UAS-Nord-HA-GFP / +* |
| Figure 8- figure supplement 1 | - *w; tub-Gal80^ts^ / UAS-GFP; hh-Gal4 / +* - *w; tub-Gal80^ts^ / UAS-GFP; hh-Gal4/ UAS-GFP* - *w; tub-Gal80^ts^ / UAS-Nord-HA-GFP; hh-Gal4 / +* - *w; tub-Gal80^ts^ / UAS-Nord-HA-GFP; hh-Gal4 / UAS-Nord-HA-GFP* |

**Supplementary file 3. The genotype of larvae, pupae or adult flies from where wing discs, pupal or adult wings were collected and imaged in each figure.**
