# Supplementary material for: The NDNF-like factor Nord is a Hedgehog-induced extracellular BMP modulator that regulates Drosophila wing patterning and growth
Source: eLife. 2022 Jan 17;11:e73357. doi: 10.7554/eLife.73357 (PMC8856659; doi:10.7554/eLife.73357)
Supplement: Supplementary file 4. [file elife-73357-supp4.docx]

**Supplementary file 4. The nucleotide sequences used to express Nord or various Ndnf fusion proteins**

pAcSV-Nord-GFP

TCTAGAGAAatgcaaaggagcaccatgttgccaggagtggagctgctgctgctgttcctgctgagcacgtcgctgcacgcggagatctcccacttgccgatggccagcaaccgcatcattctggactccacggagctgctccagaacccgctgatccaggacgccggtgtgccagcggccagggcgacgccacttccaccgcaaaagtgtccttcgctgcagcggtaccgcaagatgctaaaggacttcgacgacctggaggacctctacgtggacgtgcccgtgcatatagctctggcggagcggcagaggaagcgatttgtgctgaacctgaacgactcgacgccgctgacgattcgcttttcggcgcccgtgggccgcaagatgtactacaatcagacaggaaacccccttcggccggcggcagtggcacctgggccaggtgtggctgtgggcagtctggtgctgccctgcgccctgcgtggccagtacgacctcttcgtcctcgcccgccactcgggagccctcaaggtggacgccctggcagagcatccccagcacgactggcccctcctcaacgccacgcaccgaatcgccattcgcacccagaatcgcgtgcggaagcgcgagatgatcgtcaagtgggaaaggagcaagtttgatttccatgtcatgcactattgcctggtgattcagagactctccatggacacgccgcggatgatcttcaccaacttctgccaggcggtgtccgcctacacggaccagcagcccatgactcccagttgcgccgcaggcggtcccttggaagggatttggggtgctccaccccagcgggaaaggagacttaatcctaggcagaacgttcacatcgtgtgcacggggaagaggcgccaacagctgctgcgacgccttcttccgaggagcagttaccatctggacctgttcggaatccaccagggtagacagaacctcaccctgcgactggccagcagccaggtgaactttaaccgcacccaacctctggccctgaagcagcaggctctgatgcaactcaagatcggtggacagcacgggaagcaggtgtacagcttcaaggttccgcagacgactcgccagctgggggagccgttcatgaggcaccttttgatcccttgctcgggcagcgaaatccgggtgaagctgctacggcaacgcagcgaggtggggaaaacggaggccttctatagtccaacgtacattcggcagaagggagtcctaccaggggagcgctatctcatgcggttcgagcccagcaacgatgacgaggcgttgcgagctcagaaggtcatggtggccctgagcagcgaggctctgttccgggatttgcccgagctgccgcagaacacgacagtcttcaatgtgcgaacgcggtgcagcagagccaccattgcatggaatggctcaccggatgaacgggagctgagctactgcatcatagtgttcaacctgccgcagcgcaatcgcagcgtggtggactttaccaactactgcatggactttgtgcccaagcgggtgatgcagtacagatacttcgagtggatgacctgcagggagaggcagcagagtcccgacaacatcgagacggagaccatactgaacctgatgccgggttccagctacctggtctacgtaacggccaacctgagcatgggcaagccgctgccctaccaggcgctgaccctccacatggccagtcagtgcctggacggatcgcacgagtccttctacCTCGAGATGGTGAGCAAGGGCGAGGAGCTGTTCACCGGGGTGGTGCCCATCCTGGTCGAGCTGGACGGCGACGTAAACGGCCACAAGTTCAGCGTGTCCGGCGAGGGCGAGGGCGATGCCACCTACGGCAAGCTGACCCTGAAGTTCATCTGCACCACCGGCAAGCTGCCCGTGCCCTGGCCCACCCTCGTGACCACCTTCGGCTACGGCCTGCAGTGCTTCGCCCGCTACCCCGACCACATGAAGCAGCACGACTTCTTCAAGTCCGCCATGCCCGAAGGCTACGTCCAGGAGCGCACCATCTTCTTCAAGGACGACGGCAACTACAAGACCCGCGCCGAGGTGAAGTTCGAGGGCGACACCCTGGTGAACCGCATCGAGCTGAAGGGCATCGACTTCAAGGAGGACGGCAACATCCTGGGGCACAAGCTGGAGTACAACTACAACAGCCACAACGTCTATATCATGGCCGACAAGCAGAAGAACGGCATCAAGGTGAACTTCAAGATCCGCCACAACATCGAGGACGGCAGCGTGCAGCTCGCCGACCACTACCAGCAGAACACCCCCATCGGCGACGGCCCCGTGCTGCTGCCCGACAACCACTACCTGAGCTACCAGTCCGCCCTGAGCAAAGACCCCAACGAGAAGCGCGATCACATGGTCCTGCTGGAGTTCGTGACCGCCGCCGGGATCACTCTCGGCATGGACGAGCTGTACAAGTAA GAATTC

XXXXX Nord coding sequence

XXXXX GFP coding sequence

pAcSV-Nord-HA-GFP

TCTAGAGAAatgcaaaggagcaccatgttgccaggagtggagctgctgctgctgttcctgctgagcacgtcgctgcacgcggagatctcccacttgccgatggccagcaaccgcatcattctggactccacggagctgctccagaacccgctgatccaggacgccggtgtgccagcggccagggcgacgccacttccaccgcaaaagtgtccttcgctgcagcggtaccgcaagatgctaaaggacttcgacgacctggaggacctctacgtggacgtgcccgtgcatatagctctggcggagcggcagaggaagcgatttgtgctgaacctgaacgactcgacgccgctgacgattcgcttttcggcgcccgtgggccgcaagatgtactacaatcagacaggaaacccccttcggccggcggcagtggcacctgggccaggtgtggctgtgggcagtctggtgctgccctgcgccctgcgtggccagtacgacctcttcgtcctcgcccgccactcgggagccctcaaggtggacgccctggcagagcatccccagcacgactggcccctcctcaacgccacgcaccgaatcgccattcgcacccagaatcgcgtgcggaagcgcgagatgatcgtcaagtgggaaaggagcaagtttgatttccatgtcatgcactattgcctggtgattcagagactctccatggacacgccgcggatgatcttcaccaacttctgccaggcggtgtccgcctacacggaccagcagcccatgactcccagttgcgccgcaggcggtcccttggaagggatttggggtgctccaccccagcgggaaaggagacttaatcctaggcagaacgttcacatcgtgtgcacggggaagaggcgccaacagctgctgcgacgccttcttccgaggagcagttaccatctggacctgttcggaatccaccagggtagacagaacctcaccctgcgactggccagcagccaggtgaactttaaccgcacccaacctctggccctgaagcagcaggctctgatgcaactcaagatcggtggacagcacgggaagcaggtgtacagcttcaaggttccgcagacgactcgccagctgggggagccgttcatgaggcaccttttgatcccttgctcgggcagcgaaatccgggtgaagctgctacggcaacgcagcgaggtggggaaaacggaggccttctatagtccaacgtacattcggcagaagggagtcctaccaggggagcgctatctcatgcggttcgagcccagcaacgatgacgaggcgttgcgagctcagaaggtcatggtggccctgagcagcgaggctctgttccgggatttgcccgagctgccgcagaacacgacagtcttcaatgtgcgaacgcggtgcagcagagccaccattgcatggaatggctcaccggatgaacgggagctgagctactgcatcatagtgttcaacctgccgcagcgcaatcgcagcgtggtggactttaccaactactgcatggactttgtgcccaagcgggtgatgcagtacagatacttcgagtggatgacctgcagggagaggcagcagagtcccgacaacatcgagacggagaccatactgaacctgatgccgggttccagctacctggtctacgtaacggccaacctgagcatgggcaagccgctgccctaccaggcgctgaccctccacatggccagtcagtgcctggacggatcgcacgagtccttctacTACCCATACGACGTCCCAGACTACGCTCTCGAGATGGTGAGCAAGGGCGAGGAGCTGTTCACCGGGGTGGTGCCCATCCTGGTCGAGCTGGACGGCGACGTAAACGGCCACAAGTTCAGCGTGTCCGGCGAGGGCGAGGGCGATGCCACCTACGGCAAGCTGACCCTGAAGTTCATCTGCACCACCGGCAAGCTGCCCGTGCCCTGGCCCACCCTCGTGACCACCTTCGGCTACGGCCTGCAGTGCTTCGCCCGCTACCCCGACCACATGAAGCAGCACGACTTCTTCAAGTCCGCCATGCCCGAAGGCTACGTCCAGGAGCGCACCATCTTCTTCAAGGACGACGGCAACTACAAGACCCGCGCCGAGGTGAAGTTCGAGGGCGACACCCTGGTGAACCGCATCGAGCTGAAGGGCATCGACTTCAAGGAGGACGGCAACATCCTGGGGCACAAGCTGGAGTACAACTACAACAGCCACAACGTCTATATCATGGCCGACAAGCAGAAGAACGGCATCAAGGTGAACTTCAAGATCCGCCACAACATCGAGGACGGCAGCGTGCAGCTCGCCGACCACTACCAGCAGAACACCCCCATCGGCGACGGCCCCGTGCTGCTGCCCGACAACCACTACCTGAGCTACCAGTCCGCCCTGAGCAAAGACCCCAACGAGAAGCGCGATCACATGGTCCTGCTGGAGTTCGTGACCGCCGCCGGGATCACTCTCGGCATGGACGAGCTGTACAAGTAA GAATTCGGA

XXXXX Nord coding sequence

XXXXX GFP coding sequence

XXXXX HA tag coding sequence

pUAST-Nord-HA-GFP

TCTAGAGAAatgcaaaggagcaccatgttgccaggagtggagctgctgctgctgttcctgctgagcacgtcgctgcacgcggagatctcccacttgccgatggccagcaaccgcatcattctggactccacggagctgctccagaacccgctgatccaggacgccggtgtgccagcggccagggcgacgccacttccaccgcaaaagtgtccttcgctgcagcggtaccgcaagatgctaaaggacttcgacgacctggaggacctctacgtggacgtgcccgtgcatatagctctggcggagcggcagaggaagcgatttgtgctgaacctgaacgactcgacgccgctgacgattcgcttttcggcgcccgtgggccgcaagatgtactacaatcagacaggaaacccccttcggccggcggcagtggcacctgggccaggtgtggctgtgggcagtctggtgctgccctgcgccctgcgtggccagtacgacctcttcgtcctcgcccgccactcgggagccctcaaggtggacgccctggcagagcatccccagcacgactggcccctcctcaacgccacgcaccgaatcgccattcgcacccagaatcgcgtgcggaagcgcgagatgatcgtcaagtgggaaaggagcaagtttgatttccatgtcatgcactattgcctggtgattcagagactctccatggacacgccgcggatgatcttcaccaacttctgccaggcggtgtccgcctacacggaccagcagcccatgactcccagttgcgccgcaggcggtcccttggaagggatttggggtgctccaccccagcgggaaaggagacttaatcctaggcagaacgttcacatcgtgtgcacggggaagaggcgccaacagctgctgcgacgccttcttccgaggagcagttaccatctggacctgttcggaatccaccagggtagacagaacctcaccctgcgactggccagcagccaggtgaactttaaccgcacccaacctctggccctgaagcagcaggctctgatgcaactcaagatcggtggacagcacgggaagcaggtgtacagcttcaaggttccgcagacgactcgccagctgggggagccgttcatgaggcaccttttgatcccttgctcgggcagcgaaatccgggtgaagctgctacggcaacgcagcgaggtggggaaaacggaggccttctatagtccaacgtacattcggcagaagggagtcctaccaggggagcgctatctcatgcggttcgagcccagcaacgatgacgaggcgttgcgagctcagaaggtcatggtggccctgagcagcgaggctctgttccgggatttgcccgagctgccgcagaacacgacagtcttcaatgtgcgaacgcggtgcagcagagccaccattgcatggaatggctcaccggatgaacgggagctgagctactgcatcatagtgttcaacctgccgcagcgcaatcgcagcgtggtggactttaccaactactgcatggactttgtgcccaagcgggtgatgcagtacagatacttcgagtggatgacctgcagggagaggcagcagagtcccgacaacatcgagacggagaccatactgaacctgatgccgggttccagctacctggtctacgtaacggccaacctgagcatgggcaagccgctgccctaccaggcgctgaccctccacatggccagtcagtgcctggacggatcgcacgagtccttctacTACCCATACGACGTCCCAGACTACGCTCTCGAGATGGTGAGCAAGGGCGAGGAGCTGTTCACCGGGGTGGTGCCCATCCTGGTCGAGCTGGACGGCGACGTAAACGGCCACAAGTTCAGCGTGTCCGGCGAGGGCGAGGGCGATGCCACCTACGGCAAGCTGACCCTGAAGTTCATCTGCACCACCGGCAAGCTGCCCGTGCCCTGGCCCACCCTCGTGACCACCTTCGGCTACGGCCTGCAGTGCTTCGCCCGCTACCCCGACCACATGAAGCAGCACGACTTCTTCAAGTCCGCCATGCCCGAAGGCTACGTCCAGGAGCGCACCATCTTCTTCAAGGACGACGGCAACTACAAGACCCGCGCCGAGGTGAAGTTCGAGGGCGACACCCTGGTGAACCGCATCGAGCTGAAGGGCATCGACTTCAAGGAGGACGGCAACATCCTGGGGCACAAGCTGGAGTACAACTACAACAGCCACAACGTCTATATCATGGCCGACAAGCAGAAGAACGGCATCAAGGTGAACTTCAAGATCCGCCACAACATCGAGGACGGCAGCGTGCAGCTCGCCGACCACTACCAGCAGAACACCCCCATCGGCGACGGCCCCGTGCTGCTGCCCGACAACCACTACCTGAGCTACCAGTCCGCCCTGAGCAAAGACCCCAACGAGAAGCGCGATCACATGGTCCTGCTGGAGTTCGTGACCGCCGCCGGGATCACTCTCGGCATGGACGAGCTGTACAAGTAA GAATTCGGA

XXXXX Nord coding sequence

XXXXX GFP coding sequence

XXXXX HA tag coding sequence

pcDNA3.1-Nord-HisMyc

atgcaaaggagcaccatgttgccaggagtggagctgctgctgctgttcctgctgagcacgtcgctgcacgcggagatctcccacttgccgatggccagcaaccgcatcattctggactccacggagctgctccagaacccgctgatccaggacgccggtgtgccagcggccagggcgacgccacttccaccgcaaaagtgtccttcgctgcagcggtaccgcaagatgctaaaggacttcgacgacctggaggacctctacgtggacgtgcccgtgcatatagctctggcggagcggcagaggaagcgatttgtgctgaacctgaacgactcgacgccgctgacgattcgcttttcggcgcccgtgggccgcaagatgtactacaatcagacaggaaacccccttcggccggcggcagtggcacctgggccaggtgtggctgtgggcagtctggtgctgccctgcgccctgcgtggccagtacgacctcttcgtcctcgcccgccactcgggagccctcaaggtggacgccctggcagagcatccccagcacgactggcccctcctcaacgccacgcaccgaatcgccattcgcacccagaatcgcgtgcggaagcgcgagatgatcgtcaagtgggaaaggagcaagtttgatttccatgtcatgcactattgcctggtgattcagagactctccatggacacgccgcggatgatcttcaccaacttctgccaggcggtgtccgcctacacggaccagcagcccatgactcccagttgcgccgcaggcggtcccttggaagggatttggggtgctccaccccagcgggaaaggagacttaatcctaggcagaacgttcacatcgtgtgcacggggaagaggcgccaacagctgctgcgacgccttcttccgaggagcagttaccatctggacctgttcggaatccaccagggtagacagaacctcaccctgcgactggccagcagccaggtgaactttaaccgcacccaacctctggccctgaagcagcaggctctgatgcaactcaagatcggtggacagcacgggaagcaggtgtacagcttcaaggttccgcagacgactcgccagctgggggagccgttcatgaggcaccttttgatcccttgctcgggcagcgaaatccgggtgaagctgctacggcaacgcagcgaggtggggaaaacggaggccttctatagtccaacgtacattcggcagaagggagtcctaccaggggagcgctatctcatgcggttcgagcccagcaacgatgacgaggcgttgcgagctcagaaggtcatggtggccctgagcagcgaggctctgttccgggatttgcccgagctgccgcagaacacgacagtcttcaatgtgcgaacgcggtgcagcagagccaccattgcatggaatggctcaccggatgaacgggagctgagctactgcatcatagtgttcaacctgccgcagcgcaatcgcagcgtggtggactttaccaactactgcatggactttgtgcccaagcgggtgatgcagtacagatacttcgagtggatgacctgcagggagaggcagcagagtcccgacaacatcgagacggagaccatactgaacctgatgccgggttccagctacctggtctacgtaacggccaacctgagcatgggcaagccgctgccctaccaggcgctgaccctccacatggccagtcagtgcctggacggatcgcacgagtccttctacCTCGAGAAGCTTGGGCCCGAACAAAAACTCATCTCAGAAGAGGATCTGAATAGCGCCGTCGACCATCATCATCATCATCATTGA

XXXXX Nord coding sequence

XXXXX Myc tag coding sequence

XXXXX His tag coding sequence

pcDNA3.1-HsNDNF-HisMyc

atggtgctgctccactggtgcctgctgtggctcctgtttccactcagctcaaggacccagaagttacccacccgggatgaggaactttttcagatgcagatccgggacaaggcattttttcatgattcgtcagtaattccagatggagctgaaattagcagttatctctttagagatacacctaaaaggtatttctttgtggttgaagaagacaatactccattatcagtcacagtgacgccctgtgatgcgcctttggagtggaagctgagcctccaggagctgccagaggacaggagcggggaaggctcaggtgatctggaacctcttgagcagcagaagcagcagatcattagtgaggaaggcactgagttattctcctacaaaggcaatgatgttgagtattttatatcgtctagttccccatctggtttatatcagttggatcttctttcaacagagaaagacacacatttcaaagtatatgccaccacaactccagaatctgatcagccataccctgagttaccctatgacccaagagtagatgtgacctcactggggcgcaccacggtcactttggcctggaaaccaagccccactgcctctttgctgaaacaacccattcagtactgtgtggtcatcaacaaagagcacaatttcaaaagtctctgtgcagtggaagcaaaactgagtgcagatgatgcttttatgatggcaccgaaacctggtctggacttcagcccctttgactttgcccactttggatttccttctgataattcaggtaaagaacgcagtttccaggcaaagccttctccaaaactggggcgtcatgtctactccaggcccaaggttgatattcagaaaatctgcataggaaacaagaacatcttcaccgtctctgatctgaaacccgacacgcagtactactttgacgtatttgtggtcaacatcaacagcaacatgagcaccgcttatgtaggtacctttgccaggaccaaggaagaagccaaacagaagacagtcgagctcaaagatgggaagataacagatgtatttgttaaaaggaagggagcaaagtttctacggtttgctccagtctcttctcaccaaaaagtcaccttctttattcactcttgtctggatgctgtccaaatccaagtgagaagagatgggaaacttcttctgtctcagaatgtggaaggcattcagcagtttcagcttagaggaaaacctaaagctaaatacctcgttcgactgaaaggaaacaagaaaggagcatctatgttgaaaattctagctaccacaaggcctactaagcagtcatttccctctcttcctgaagacacaagaatcaaagcctttgacaagctccgtacctgttcctcggccaccgtggcttggctaggcactcaggaaaggaacaagttttgcatctacaaaaaagaagtggatgataactacaatgaagaccagaagaaaagagagcaaaaccaatgtctaggaccagatataaggaagaagtcagaaaaggtcctctgtaaatatttccacagtcaaaacctgcagaaagcagtgaccacagaaacaattaaaggtcttcagcctggcaaatcttacctgctggatgtttatgtcataggacatggggggcactctgtgaagtatcagagtaaggttgtgaaaactagaaagttctgtAAGCTTGGGCCCGAACAAAAACTCATCTCAGAAGAGGATCTGAATAGCGCCGTCGACCATCATCATCATCATCATTGA

XXXXX HsNDNF coding sequence

XXXXX Myc tag coding sequence

XXXXX His tag coding sequence

pcDNA3.1-CeNdnf-HisMyc

atgctcaaactccttttgctcctttttactatccaattatgcacaccattccacgtggttcccatgagctactcgattttgtcggagaacactgaaagtgtcatcgatttggccggaaaagttagcgagaagagattctaccttcacattaccaatgaagcttccccattttttatgtatgttacgccgtgcggagctgcggttcactggcaacttttcacaatggataaggatattagcatgtctgaagagttcgatactatttccgacatgtcaaattcgctggaagaatctgaaaaattcaagttgatcgccggagaggatgataagaagagaatgacgttttttgcgcatagtttgccgaaaaaagtgatcctggtggtccgtgcggcgtcaacctcctccgcttccgtccgaatcttcttcaccccatccctgttccgtctcgaagatcaatacccaccacttccacatgacacccgtcttgccacaaacataatctccgagagtacctattccagaagggatgaggtctccacactgatcacatggaaaatctcaccgcaagttcggaacgccgagccgggtcgctaccgtatctgtacgattgtttcgcgtcgagacccagaattcgcgggaatgtgtgaccacgtggaagaaggtgtggagactgtcaaatgtgtcccacagacaaataataccgtagtcatcggctcgttgagaagagatcggacatattttgtgacggtttttgtgagggatcacaagaggggtacatcgtcggcgtatgaggtacagacgattcaaacgtcggaagttgtgacacacaggagaaaagttacccgaaatgtgccgagaaagcagaaaaagtcggctccaagagttttgagcaacgggcagctggagcaggtggagctggagccgaagaaggggacttttgtgaatttgaagtttttcgtgaatagcctgccggagggagccgagaataatacacaatcggcaatgctcattgtgcacgcttgcgatggactcgtacgtatcaatttgtttcggaacggaaaaattctcaagagaagcgacgcgttttccgggtttagaagattcgtagtcaccaacatccgcagcggacatctccgtttccaaatcgtaaacgacgacgaaactccaaaaactatcagagtctgggcgagcaccgatctcaccacgtcaccatacccaaatcttccagatgataccagcgtaaagattgtcgggcgctcgtgctcttctgcctcaattcaatggattcgtgctcacgatagtcatgtaaagtactgtgtttatagaagaagagaacattcaaattttttggaacatcttgttagtttggccgacaatctctgcgaaggcggcctctcctcatccgtactcgtcggctgctacacccattctccgacctcttccgaagacgatgcccaatccctgattgagacaacaatcggaggtcttcttccagctagtacctaccgtttagacctcctggccactccactagaccgcccgaatgctcaggctttacccttccgaactgtctgggttcggaccaatcgattctgtggagttcataatAAGCTTGGGCCCGAACAAAAACTCATCTCAGAAGAGGATCTGAATAGCGCCGTCGACCATCATCATCATCATCATTGA

XXXXX HsNDNF coding sequence

XXXXX Myc tag coding sequence

XXXXX His tag coding sequence

pcDNA3.1-DrNdnf-HisMyc

atgacgtggaggtgtggatgttatggtttggtgttgctggttttaggggtgatgggacagaaattgcccacgcgtgatgaaggcctctttcagatgcagatcagggataaagctcagtttcacgattcatctgtcatgccggatggagcggagatcagcggatacttgttcagggatacacccaaaaggtactattttgtggtggaggaagacaacactcctctgctggtgacggtgacgccctgcgatgctcctctggagtggagattgacgttgcaggagctcccggaggaccgcagcggggaaggatcaggtgagccagaaccactggaacagcagaaacagcaggtcacggcaaacgagggtaccgagctctttacctacaaaggaaatgatgtcgaatcattcatctcctccagctccccgtcaggcttgtaccagctggaaatcatctccaccgaaaaggacagcaacttcaagatttattctaccacaactcccgagtccgatcaaccataccctgagctgccatacgaccccagggtggatgttaccgcattgggtcgtactactgttacactagcttggaagcctactcctactggctcagttatgggccagccgatccagtactgtgtggttattaacaaggagcacaacttcaagagcttatgtgcagcggaggccaagatgagcctggatgatgccttcatgatagctccgaaacccggccgggactttagcccctttgactttgcatattttggttttgtcccatctgagaacgatttccataaagatcgtttcctcaccaccaacagagctctgagcaacaaaatgagtcgcgcatatatccccaagcccaaagtggccgacatacagaagatctgcataggcaacaagaatatttttaccataacggacctaaagccagacacacaatattatttcgatgtgttcgctgtcaacacaggtaccaacatgagtactgcgtatgtgggcacttttgctcgcaccaaggaagaggccaagcagaaaaccgtggagctcaaggacgggaaagtaactgatgtcttcgttaagagaaaggggagtaagttcctccgctttgcaccggtctcctctcaccagcgggtcacgctgtttgtgcatgcttgcttggatgcagtgcaggtacaggtgcggcgagatgggaaacttgttctctctcagaatgtggagggagtccgtcagttccagcttcggggaaagcccaaagccaagtacctgattcgtctgcgtgggtcccgcaaaggtgcctccacattgaaagtcctagccagtactcgtgccggaggaaagcaaccgttccctgctctgcccgaggatacccgtatcaaagctttcgataagttgcgcacctgttcttcagtcaccgtggcttggcttggcacacaggagcgcaacaagtactgtgtgtacagacgggaagtttccgaaagttacggcgaagagcatcggcgccgtgagcagaaccaatgctctggaccagagagtcgtcgcaagtctgagaaagtgctttgcaagtacttctacagcgccaaccttcagaaagctgtcacaacggagaccataacaggccttgaggcgggaaagagctacctcctggatgtttatgtggtaggacacagcggccactcagtcaagtaccagagcaaactggtgaaaacgaggaagtactgtAAGCTTGGGCCCGAACAAAAACTCATCTCAGAAGAGGATCTGAATAGCGCCGTCGACCATCATCATCATCATCATTGA

XXXXX HsNDNF coding sequence

XXXXX Myc tag coding sequence

XXXXX His tag coding sequence

pcDNA3.1-XtNdnf-HisMyc

atgaagctgtgtcgctggtacattgctctgttcctgctgccagtctgtttgcagtcccagaagttacctaccagggacgaagagctctttcagatgcagatcagagacaaggctcttttccatgactcttctgttattccagatggagctgagatcagtggctatctgtttagagacaatcctaaaagatatttctttgtagtggaagaggataatacacctttatcagtgattgtcacaccatgtgatgcaccgttggaatggaagttgactctgcaggaactgcctgaagaagccagtggagaaggatcaggtgaacctgaaccactcgaggagcaaaagcagcagattgtgaatgaagaaggcacagagttgttctcttataaaggaaatgatgtggaatattttgtatcatcaagttccccttctggcttgtatcagctggagttgatatccacagaaaaagacactcatttcaaagtctatgcaacaacaactccagagtcagaccagccgtatccagaattgccatatgatcctagagttgatgtgacatcccttgggcgcactacaatcactttagcctggaaacctacaccaacttcttctgtcatgaagcaacccattcagtactgtgttgtcatcaacaaggagcacaattttaaaagtatctgcgcagttgaagccaaaatgattgcagacgatgcatttatgatggcacctaagcctggaatagactttaacccctttgactttgctcattttggttttcaatcagataacagtgctggaaaggatcggaacttcatgcctaaagtctcctcttctaagatgttaaggcagataaccaaaccaaaagtggatatccaaaagttttgcatagggaacaaaaatatatttacagtgtccgacctgaagcctgatactcagtattactttgatgtctttgctgtcaatgctgttacaaatatgagcacagcgtatgttggaacttttgctcgaaccaaagaagaagccaagcaaaaaaccgtggaattaaaggatggcaaagtcactgatgtattcatcaagagaaagggtacaaagtttttaaggttttctccagtttcttctcaccagaaggtaacattttctgttcattcttgcctggacgccatccagatccaagtcagaagagatggaaagctcctcttgtcacagagtgttgaaggtgtacgtcagtttcagctaagagggaaacccaaagcgaaatacttaatcaggctcaagggcagcaaaaaaggtgcatcaatgctaaaaattttggcaacatccaagtttaacaaacagccattcccttcccttcctgaagacacacgaatcaaagcctttgacaaactgcgtacttgctcctctgccactgtagcatggctgggcacccaagaaagaaataaattctgcatttacaaaaaagaagtcgatgacgattacacagaggaacacaaaaaacgagaccaaaatcagtgtttgggaccagacaccagaaagaaatcagaaaaggttctctgcaaatacttccacagccagaatatccacaaagcagtcaccacagagacaattaaggggcttcagcctgggaaatcgtacatgttagacgtttacgtcatgggtcacgccggccactcagtcaaatatcaaagtaaaattgtaaagacaagaaagttctgtAAGCTTGGGCCCGAACAAAAACTCATCTCAGAAGAGGATCTGAATAGCGCCGTCGACCATCATCATCATCATCATTGA

XXXXX HsNDNF coding sequence

XXXXX Myc tag coding sequence

XXXXX His tag coding sequence

pBRAcpA-Dpp-FLAG

atgcgcgcatggcttctactcctcgcagtgctggcgacttttcaaacgattgttcgagttgctagcaccgaggatatatcccagagattcatcgccgccatagcgcccgttgccgctcatattccgctagcatcagcatcaggatcaggatcaggacgatctggatctagatcggtaggagcctcgaccagcacagcattagcaaaagcatttaatccattcagcgagcccgcctcgttcagtgatagtgataaaagccatcggagtaaaacaaacaaaaaacctagcaaaagtgacgcgaaccgacagttcaacgaagtgcataagccaagaacagaccaattagaaaattccaaaaataagtctaaacaattagttaataaacccaaccacaacaaaatggctgtcaaggagcagaggagccaccacaagaagagccaccaccatcgcagccaccagccaaagcaggccagtgcatccacagaatctcatcaatcctcgtcgattgaatcaatcttcgtggaggagccgacgctggtgctcgaccgcgaggtggcctccatcaacgtgcccgccaacgccaaggccatcatcgccgagcagggcccgtccacctacagcaaggaggcgctcatcaaggacaagctgaagccagacccctccactctagtcgagatcgagaagagcctgctctcgctgttcaacatgaagcggccgcccaagatcgaccgctccaagatcatcatccccgagccgatgaagaagctctacgccgagatcatgggccacgagctcgactcggtcaacatccccaagccgggtctgctgaccaagtcggccaacacagtgcgaagttttacacacaaagatagtaaaatcgacgatcgatttccgcaccaccatcggtttcggctgcacttcgacgtgaagagcattcccgccgacgagaagctgaaggcggcggagctgcagctgacccgggacgcactcagtcaacaggtggtggccagcagatcgtcggcgaatcggacgcgctaccaggtgcttgtctacgacatcacgcgcgtcggggtgcgtggtcagcgggagccgagctatctgctgttggacaccaagacggtccggcttaacagcacggacacggtgagcctcgatgtccagccggccgtggaccggtggctggcgagtccgcagcgcaactacggactgctggtggaggtgcggacggtccgctccctgaagccggccccacaccatcacgtacgcctgcgccgcagcgcggacgaggcgcacgagcggtggcagcacaagcagccgctcctgttcacctacacggacgacgggcggcacaaggcgcgctccattcgggacgtgtctggcggagagggcggtggcaagggcggccggaacaagcggcagccgagacggcctacgaggcgcaagaaccacgacgacaTGGAGCAAAAGCTAGCCGATTATAAGGACGATGACGACAAGCTTATTTCTGAAGAGGACTTGAATAcctgccggcggcactcgctgtacgtggacttctcggacgtgggctgggacgactggattgtggcgcctctgggctacgatgcgtactactgccacgggaagtgccccttcccgctggccgaccacttcaactcgaccaatcacgccgtggtgcagaccctggtcaacaatatgaatcccggcaaggtgccgaaggcgtgctgcgtgcccacgcaactggacagcgtggccatgctctatctcaacgaccaaagtacggtggtgctgaagaactaccaggagatgaccgtggtgggctgtggctgtcgatag

XXXXX Dpp coding sequence

XXXXX Flag coding sequence

pBRAcpA-Gbb-FLAG

atgtcgggactgcgaaacacctcggaggccgttgcagtgctcgcctccctgggactcggaatggttctgctcatgttcgtggcgaccacgccgccggccgttgaggccacccagtcggggatttacatagacaacggcaaggaccagacgatcatgcacagagtgctgagcgaggacgacaagctggacgtctcgtacgagatcctcgagttcctgggcatcgccgaacggccgacgcacctgagcagccaccagttgtcgctgaggaagtcggctcccaagttcctgctggacgtctaccaccgcatcacggcggaggagggtctcagcgatcaggatgaggacgacgactacgaacgcggccatcggtccaggaggagcgccgacctcgaggaggatgagggcgagcagcagaagaacttcatcaccgacctggacaagcgggccatcgacgagagcgacatcatcatgaccttcctgaacaagcgccaccacaatgtggacgaactgcgtcacgagcacggccgtcgcctgtggttcgacgtctccaacgtgcccaacgacaactacctggtgatggccgagctgcgcatctatcagaacgccaacgagggcaagtggctgaccgccaacagggagttcaccatcacggtatacgccattggcaccggcacgctgggccagcacaccatggagccgctgtcctcggtgaacaccaccggggactacgtgggctggttggagctcaacgtgaccgagggcctgcacgagtggctggtcaagtcgaaggacaatcatggcatctacattggagcacacgctgtcaaccgacccgaccgcgaggtgaagctggacgacattggactgatccaccgcaaggtggacgacgagttccagcccttcatgatcggcttcttccgcggaccggagctgatcaaggcgacggcccacagcagccaccacaggagcaagcgaagcgccagccatccacgcaagcgcaagaagtcggtgtcgcccaacaacgtgccgctgctggaaccgatggagagcacgcgcagcGATTATAAGGATGACGACGACAAGCTTCTGGAACCGATGGAGAGCACGCGCAGCtgccagatgcagaccctgtacatagacttcaaggatctgggctggcatgactggatcatcgcaccagagggctatggcgccttctactgcagcggcgagtgcaatttcccgctcaatgcgcacatgaacgccacgaaccatgcgatcgtccagaccctggtccacctgctggagcccaagaaggtgcccaagccctgctgcgctccgaccaggctgggagcactacccgttctgtaccacctgaacgacgagaatgtgaacctgaaaaagtatagaaacatgattgtgaaatcctgcgggtgccattga

XXXXX Dpp coding sequence

XXXXX Flag coding sequence

pBRAcpA-Gbb-HA

atgtcgggactgcgaaacacctcggaggccgttgcagtgctcgcctccctgggactcggaatggttctgctcatgttcgtggcgaccacgccgccggccgttgaggccacccagtcggggatttacatagacaacggcaaggaccagacgatcatgcacagagtgctgagcgaggacgacaagctggacgtctcgtacgagatcctcgagttcctgggcatcgccgaacggccgacgcacctgagcagccaccagttgtcgctgaggaagtcggctcccaagttcctgctggacgtctaccaccgcatcacggcggaggagggtctcagcgatcaggatgaggacgacgactacgaacgcggccatcggtccaggaggagcgccgacctcgaggaggatgagggcgagcagcagaagaacttcatcaccgacctggacaagcgggccatcgacgagagcgacatcatcatgaccttcctgaacaagcgccaccacaatgtggacgaactgcgtcacgagcacggccgtcgcctgtggttcgacgtctccaacgtgcccaacgacaactacctggtgatggccgagctgcgcatctatcagaacgccaacgagggcaagtggctgaccgccaacagggagttcaccatcacggtatacgccattggcaccggcacgctgggccagcacaccatggagccgctgtcctcggtgaacaccaccggggactacgtgggctggttggagctcaacgtgaccgagggcctgcacgagtggctggtcaagtcgaaggacaatcatggcatctacattggagcacacgctgtcaaccgacccgaccgcgaggtgaagctggacgacattggactgatccaccgcaaggtggacgacgagttccagcccttcatgatcggcttcttccgcggaccggagctgatcaaggcgacggcccacagcagccaccacaggagcaagcgaagcgccagccatccacgcaagcgcaagaagtcggtgtcgcccaacaacgtgccgctgctggaaccgatggagagcacgcgcTATCCATATGATGTTCCTGATTATGCTagctgccagatgcagaccctgtacatagacttcaaggatctgggctggcatgactggatcatcgcaccagagggctatggcgccttctactgcagcggcgagtgcaatttcccgctcaatgcgcacatgaacgccacgaaccatgcgatcgtccagaccctggtccacctgctggagcccaagaaggtgcccaagccctgctgcgctccgaccaggctgggagcactacccgttctgtaccacctgaacgacgagaatgtgaacctgaaaaagtatagaaacatgattgtgaaatcctgcgggtgccattga

XXXXX Gbb coding sequence

XXXXX HA tag coding sequence

pBRAcpA-Mad-Flag

atgGACTACAAGGACGACGATGACAAGgacaccgacgatgtggaatcgaacacaagcagcgcgatgtccacactgggctcgctattctccttcacatcgccggcggtgaagaagctgctgggctggaaacagggtgacgaagaggagaagtgggcggagaaggccgtcgacagtctggtgaagaagttgaagaagcgcaagggcgccatcgaggagctggagcgtgcgctctcctgtcccggtcagccctcgaagtgtgtcaccattccacgctcgctggacggacgattacaggtctcccatcgcaagggtctgccgcatgtgatctactgccgcgtgtggcgctggccagatctgcagtcgcaccacgaactgaagccactcgagctgtgccagtatccgttcagcgccaagcagaaggaggtgtgcatcaatccgtatcactataagcgcgtggagagtccggtgctcccgccagtactcgttcctcgccactcggaattcgcgcccggtcactcgatgctgcagttcaaccatgtggccgagcccagtatgccgcacaatgtgagctactcgaacagtggattcaactcgcacagcttgagcaccagcaacacatcggtgggcagtccgagttccgtcaactccaatcccaattcgccgtacgacagcttggcgggaacaccgccacccgcctacagtccctcggaggatggcaactccaacaatccgaacgacggtggccagctgctggatgctcagatgggcgatgttgcccaggttagctattcggagcccgccttctgggcgtcgatagcctactacgagctaaattgccgcgtgggcgaggtgttccactgcaacaacaactccgtgatcgttgacggtttcacgaatccttccaacaactcggaccgctgctgtctcggccagctgagcaacgtgaacaggaatagcaccatcgagaacacacgccgtcatataggcaagggtgttcatttatactatgtgaccggcgaggtctacgccgaatgcctatccgactccgccattttcgtgcagtcgcgcaactgcaactaccaccacggattccatccgagcaccgtgtgcaaaataccgccgggctgctcgctgaagatcttcaacaatcaggaatttgctcagctgctgtcgcagtcggtgaacaatggattcgaggccgtctacgagctgacaaagatgtgcacgatccggatgtcgttcgtgaagggctggggtgcggagtatcatcgccaggatgtgacctcgacgccctgttggattgaaatccatctgcacgggccgctccagtggctggacaaggtgctcacccaaatgggctctccgcataatgcaattagttcggtatcctaa

XXXXX Mad coding sequence

XXXXX Flag coding sequence
